# Effect of Connectivity on the Carrier Transport and Recombination Dynamics of Perovskite Quantum-Dot Networks

**DOI:** 10.1021/acsnano.3c10239

**Published:** 2024-01-11

**Authors:** David O. Tiede, Carlos Romero-Pérez, Katherine A. Koch, K. Burak Ucer, Mauricio E. Calvo, Ajay Ram Srimath Kandada, Juan F. Galisteo-López, Hernán Míguez

**Affiliations:** † Instituto de Ciencias de Materiales de Sevilla (Consejo Superior de Investigaciones Científicas-Universidad de Sevilla), C/Américo Vespucio, 49, Sevilla 41092, Spain; ‡ Department of Physics and Center for Functional Materials, Wake Forest University, 1834 Wake Forest Road, Winston-Salem, North Carolina 27109, United States

**Keywords:** Semiconductor quantum-dot networks, halide perovskites, trap states, lifetime, carrier recombination

## Abstract

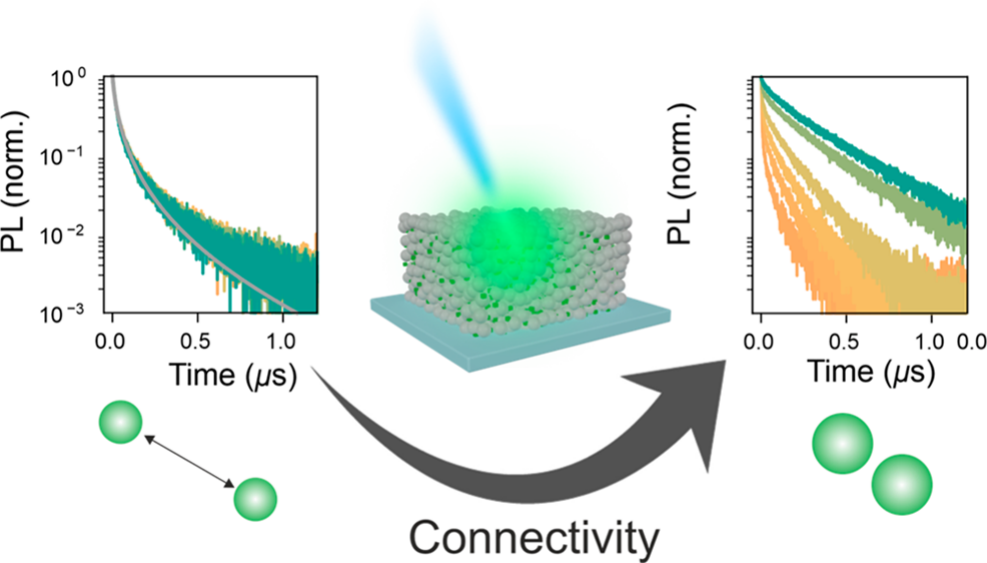

Quantum-dot (QD) solids are being widely exploited as a solution-processable technology to develop photovoltaic, light-emission, and photodetection devices. Charge transport in these materials is the result of a compromise between confinement at the individual QD level and electronic coupling among the different nanocrystals in the ensemble. While this is commonly achieved by ligand engineering in colloidal-based systems, ligand-free QD assemblies have recently emerged as an exciting alternative where nanostructures can be directly grown into porous matrices with optical quality as well as control over their connectivity and, hence, charge transport properties. In this context, we present a complete photophysical study comprising fluence- and temperature-dependent time-resolved spectroscopy to study carrier dynamics in ligand-free QD networks with gradually varying degrees of interconnectivity, which we achieve by changing the average distance between the QDs. Analysis of the photoluminescence and absorption properties of the QD assemblies, involving both static and time-resolved measurements, allows us to identify the weight of the different recombination mechanisms, both radiative and nonradiative, as a function of QD connectivity. We propose a picture where carrier diffusion, which is needed for any optoelectronic application and implies interparticle transport, gives rise to the exposure of carriers to a larger defect landscape than in the case of isolated QDs. The use of a broad range of fluences permits extracting valuable information for applications demanding either low- or high-carrier-injection levels and highlighting the relevance of a judicious design to balance recombination and diffusion.

The potential of semiconductor QDs for optoelectronic applications is dictated by the electronic coupling between them when they are assembled into device compatible QD solids. Here, beyond the possibility of tuning the optical and electronic properties of individual QDs by acting on their size, composition, and ligand structure, one can also exploit the properties arising from collective interactions.^1^ Controlling the QD separation, effective electronic and energy transport in devices can be attained while maintaining the characteristic quantum size confinement features. This has in fact already resulted in impressive advances in photovoltaic^2^ as well as electrically pumped solution-processed lasers.^3^ Within this context, over the past decade, lead halide perovskite QDs have emerged as an exciting alternative in the field of QD optoelectronics.^4^ Together with the possibility of tuning their electronic properties by controlling their size, halide perovskite QDs have an additional degree of freedom in their composition where employing a combination of two halides (Cl/Br or Br/I) allows for tuning their electronic bandgap across the visible and near-infrared spectral regions.^5,6^ In this direction, perovskite optoelectronic devices containing QD films as active layers, such as solar cells,^7^ LEDs,^8^ or photodetectors,^9^ have recently demonstrated outstanding performances in line with or surpassing other semiconductor QD-based devices. As with the well-established II–VI and III–V inorganic semiconductor QDs, colloidal synthesis in its various forms^4^ has become the main synthetic route for perovskite QDs. Within this approach, interparticle spacing could be tuned by varying the ligand employed,^10,11^ which adds complexity to the analysis of collective effects, since each ligand may give rise to different transport properties.^12−14^ Alternatively, confinement can be achieved by synthesizing QDs within porous matrices, where the interparticle distance is controlled by the concentration of nanocrystals, which is in turn determined by the porosity of the host matrix and the precursor concentration.^15−20^ This approach permits the synthesis of highly emitting and stable perovskite QDs directly into thin films with optical quality amenable to being introduced as an active layer within an optoelectronic device.^17,21^ Central to the analysis presented herein, these nanostructures allow studying the photophysical properties of nanostructures isolated from any ligand- or solvent-induced effect.^22^

In this work, we employ ligand-free perovskite QDs embedded within the pores of metal oxide nanoporous films as a test bench to understand the different collective phenomena leading to charge carrier recombination in a QD solid. By tuning the average inter-QD separation, we are able to controllably modulate the interaction between them and move from a scenario of isolated QDs to one of interconnected QDs where collective phenomena dictate the optoelectronic properties of the network. Time-resolved spectroscopic techniques are employed to study charge carrier recombination and unveil the gradually increasing influence of interconnectivity as the average interparticle distance is controllably reduced. We explore different regimes from those comprising low carrier densities, where recombination is limited by defect-assisted processes relevant for photovoltaic applications, to those comprising large carrier densities, where many-particle processes come into play, a scenario characteristic of light emitting devices. Results reveal that dot-to-dot transport and diffusion result in the exposure of the carriers to a larger defect landscape, which determines the efficiency of the different recombination processes occurring in the ensemble.

## Results and Discussion

### Structure and Linear Optical Response of Ligand-Free Perovskite QD Networks

**Figure 1:**
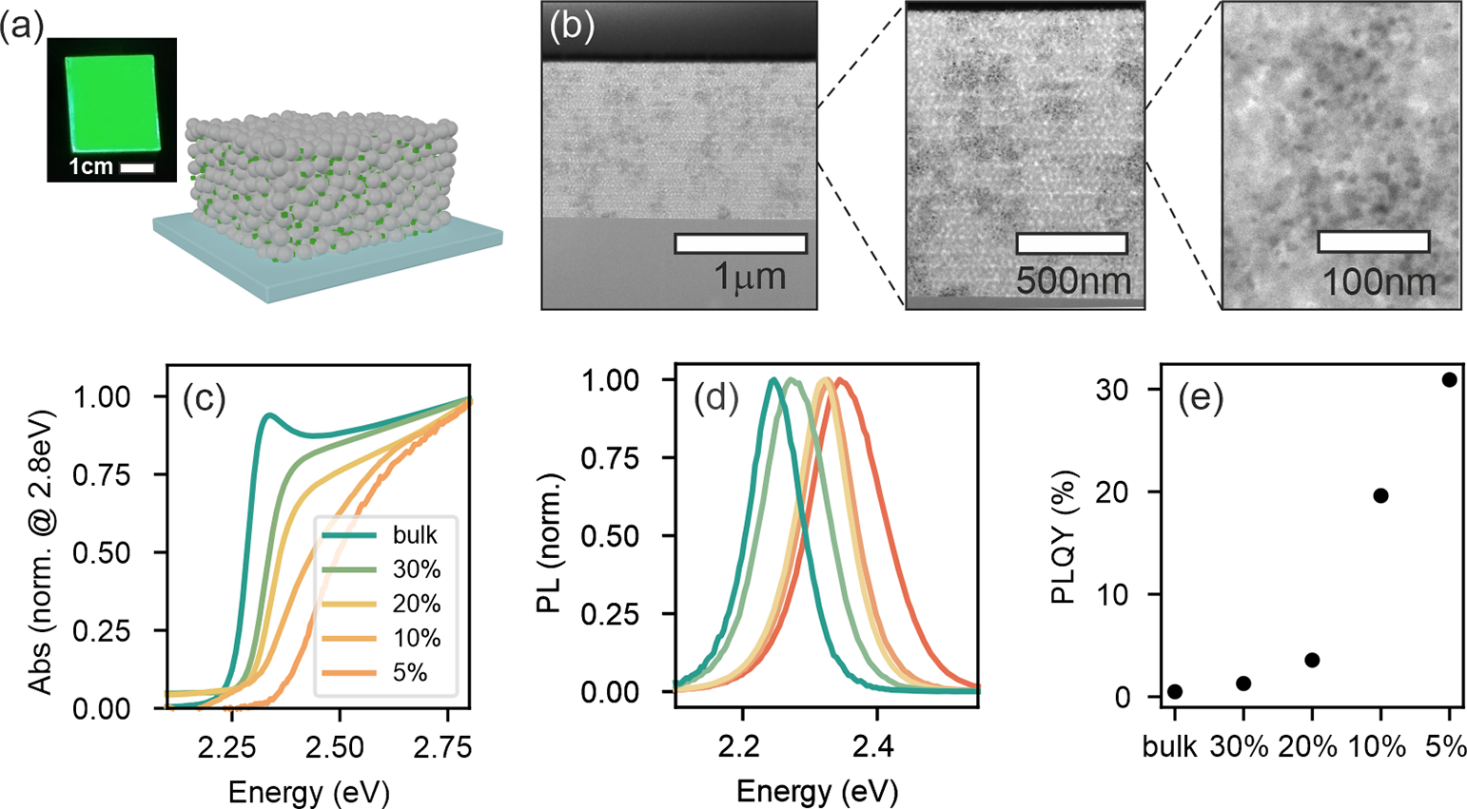
Structural and linear optical characterization of ligand-free perovskite QDs embedded in porous matrices. (a) Sketch of the analyzed materials system with an optical photograph of the thin film. (b) TEM cross section of *C*_*prec*_ = 20% at different magnifications. (c) Linear absorption normalized at 2.8 eV. (d) Normalized PL values of bulk and QD samples. The color code is the same as that in (c). (e) PLQY of the different samples shown in parts (c) and (d).

Ligand-free formamidinium lead bromide (FAPbBr_3_) QDs were synthesized within nanoporous SiO_2_ matrices by spin-coating a solution of perovskite precursors in DMSO into the matrices, following a procedure thoroughly described elsewhere.^23^ Upon annealing at 100 °C, perovskite QDs form in the voids of the SiO_2_ matrix, as illustrated in Figure 1a, where the precursor concentration *C*_*prec*_ defines the overall amount of material that is injected into the host system. Figure 1b shows a transmission electron microscopy (TEM) micrograph of an intermediate concentration, which illustrates the dispersion of crystallites attained in the matrix and their characteristic size distribution. The average crystal size, the number of particles formed per unit volume of the pores (or filling fraction, *ff*), and the average interparticle distance are affected by *C*_*prec*_ (see Table 1), allowing tuning of the interconnectivity without affecting their chemical composition, morphology, or surface properties. All samples show clear signatures of quantum confinement effects, as evidenced by the shift toward higher energies of both the absorption edge (Figure 1c) and emission spectrum (Figure 1d) as the average QD radius, ⟨*r*⟩, is reduced,^20^ although contributions to this shift from the dielectric environment cannot be discarded.^24,25^ A reduction in the QD size also leads to an enhancement in the continuous wave photoluminescence quantum yield (cw-PLQY) (Figure 1e). While this is the expected trend for an increasing level of quantum confinement, the key factor determining emission yield will be dictated by the average interparticle distance, as discussed below. It should be noticed that the excitonic peak at the absorption edge, clearly evident for the bulk case, is absent for the QDs under study. Such behavior has been found in QD solids in the past and associated with the presence of interdot electronic coupling and/or size polydispersity.^26,27^ The key structural parameters, ⟨*r*⟩ and *ff*, are obtained by fitting the spectral position of the PL maximum to the Brus equation^28^ and performing inductively coupled plasma (ICP) measurements, respectively. The Brus equation, while bearing a number of uncertainties due to the assumptions made (see Section S1.1 in the SI for a full discussion), has been proven to yield a good estimate of ⟨*r*⟩ for similar samples.^29^Table 1 shows the estimated average size, which lies always below the Bohr radius for this material (8 nm).^30^ Both ⟨*r*⟩ and *ff* values are used to estimate the average spacing between the embedded QDs, *d*_*QD*_ as summarized in Table 1 (see the SI for details on the calculations). Please note that the infiltrated system we are dealing with shows heterogeneities as a result of a tendency of QDs to cluster in certain regions of the matrix, as is evident in the TEM images shown in Figure 1b. Hence, interconnectivity can only be reached when these clusters coalesce, allowing charge percolation, which occurs above a certain QD load. In this context, the mean QD separation *d_QD_* should be taken only as a quantitative label that is inversely proportional to the degree of connectivity. The data in Table 1 clearly show that while changing *C_prec_* modifies the QD size, the main effect is on the filling fraction and therefore on the average separation between QDs, *d_QD_*, thus allowing control on interdot connectivity.

**Table 1:** Precursor Concentration, Fill Fraction, Estimated Average QD Radius, and Average Interparticle Spacing Extracted from ICP and Optical Measurements

*C*_*prec*_	*ff*	⟨*r*⟩	*d*_*QD*_
5%	0.0141	4.5 nm	28 nm
10%	0.0405	5.7 nm	22 nm
20%	0.092	6.3 nm	15 nm
30%	0.145	6.7 nm	12 nm

### Trap Filling and Carrier Recombination Dynamics in Isolated and Interconnected Perovskite QD Networks

**Figure 2:**
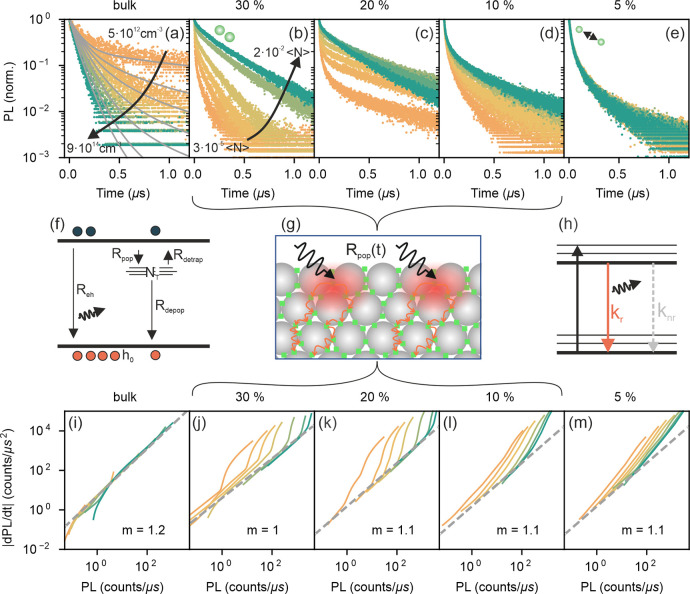
TRPL measurements and corresponding charge carrier recombination models. (a–e) Fluence-dependent TRPL measurements for bulk and QD samples with different *C*_*prec*_. Maximum and minimum fluences are indicated as carrier densities (or average excitations per QD) for the bulk (or QD) cases. Gray solid lines in (a) indicate fits to the SRH model, while the gray line in (e) corresponds to the fit to a log-normal distribution of decay rates. Insets in (b) and (e) illustrate the expected crystal size and spacing (not to scale). (f) Model of the electron transition processes considered within the SRH model. (g) Drawing exemplifying the reduced diffusivity caused by the limited interconnection of QDs. (h) Model of an isolated emitter excitation and recombination. (i–m) Derivative of emitted photon flux against photon flux for the same samples in (a–e).

To understand the role of QD interconnectivity on the charge carrier recombination, we start by studying the dynamics of the PL measured from the set of samples described above, which show a gradually varying interparticle distance *d*_*QD*_. Results are presented in Figure 2a–e. The fluence conditions used in this experiment can be considered to be on the lower end, with an average of 10^–3^–10^–1^ excitations per QD. For the case of a reference FAPbBr_3_ bulk sample (Figure 2a), we obtain long PL lifetimes on the order of μs, which are strongly fluence-dependent. The Shockley–Read–Hall (SRH) model^31,32^ can be applied to this scenario, where the recombination dynamics depends on the global population density of charge carriers that can freely move and distribute within the excited area, which is limited by the density of carriers trapped at defects. Here, the experimentally measured time-resolved photoluminescence (TRPL) dynamics depends on the macroscopic density of electrons *n*_*e*_, electron holes *n*_*h*_, and trapped carriers *n*_*T*_, and their recombination dynamics can be described by coupled rate equations (see Section S2.1 in Supporting Information), which account for the processes schematized in Figure 2f. This model is well-established for perovskite bulk samples^33−35^ and yields a perfect fit of the TRPL dynamics of the bulk reference (gray solid lines in Figure 2a), which provides defect densities and recombination rates in order with those reported in the literature (see fit values in Section S2.1 of the SI). This evidences that charge recombination under these conditions is dominated by trap filling and detrapping dynamics. Interestingly, the fluence-dependence of the TRPL in the QD films becomes less pronounced as the filling fraction in the porous films decreases and is eventually lost for the samples with the lowest amount of perovskite in the pore (*C*_*prec*_ = 5%, i.e., those with the largest *d*_*QD*_). In what follows, we analyze these dynamics in more detail.

For the cases of sparsely separated QDs , Figure 2b–d), the TRPL dynamics retains a fluence-dependent behavior, albeit with markedly distinct trends with respect to the bulk sample. The PL decays now present two components: a fast (tens of ns) and intense initial decay that can be associated with redistribution effects followed by a much slower (several μs long decay) due to trap-assisted recombination dynamics. This assignment will become more evident in the analysis of the results presented in Figure 2i–m. As the fluence increases, the second component becomes more rapid and dominant in amplitude. Notably, the SRH model that has been successful in the bulk case fails to yield a good fit to these PL dynamics, indicating a scenario more complex than that depicted in Figure 2f. For the extreme case of the largely separated QDs , the TRPL dynamics turns fluence-independent (Figure 2e). This can be interpreted in terms of isolated QDs where excitations are now well-localized, and dot-to-dot charge transport is not allowed. In this isolated QD scenario, the TRPL lifetime is determined by the ratio between nonradiative and radiative recombination pathways, characterized by rate constants *k*_*nr*_ and *k*_*r*_, respectively (Figure 2h). For a homogeneous distribution of monodisperse QDs, a single exponential decay is the expected characteristic of excitonic monomolecular recombination.^36^ However, in our case (Figure 2e), the TRPL presents a clear multiexponential behavior, which is best fitted considering a log-normal distribution of decay rates. While this can be attributed to the QD size and shape polydispersity present in practically all collections of QDs, in our case, the contribution from a potentially different defect density or strain cannot be ruled out (see Section S2.2 and Figure S2.2 in the SI). Here, it must be noted that the presence of a certain degree of size dispersion in our QD arrays will lead to funneling during charge recombination in the concentrated samples, where interconnectivity is expected to be higher. In this situation, emission will come from the largest QDs, and TRPL should be independent of the spectral region under consideration (see Figure S2.2i,j).

The effect of QD interconnectivity can be directly visualized and further analyzed by plotting the derivative of the emitted photon flux (PL in counts per second) as a function of the emitted photon flux (Figure 2i–m). This derivative can be interpreted as proportional to an instantaneous effective carrier lifetime , similar to the differential lifetime introduced by Kirchartz in ref (37), which bears information on the predominant recombination mechanism at a given time or carrier density (see below). Such a representation on the log–log scale is shown in Figure 2i–m for all samples at all excitation densities employed. In order to interpret these graphs, it should be considered that, in a quasiequilibrium condition and assuming the photophysical model depicted in Figure 2f, the first derivate of the photoluminescence can be written as (see Section S2.3):
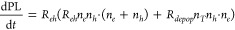
1

Considering that the emitted photon flux is proportional to the product of the free electron and hole densities (*n*_*e*_*n*_*h*_), eq 1 implies that the time variation of the photon flux is directly dependent on the total photon flux, and hence, the curves for all different excitation conditions must all line up in the log–log representation. Also, a monomolecular decay should result in a linear dependence of the  vs. PL curve with a slope close to 1, while a bimolecular one would yield a slope of around 1.5 (see discussion in SI Section S2.3 for more details). With this in mind, we can straightforwardly conclude that the PL decay dynamics of the bulk perovskite, shown in Figure 2i, is mainly dominated by trap-limited monomolecular recombination (slope *m* = 1.1), with an effective lifetime, , that depends only on the initial excitation density. On the other hand, a similar analysis of the curves plotted in Figure 2j–l reveals that the emitted photon flux does not follow a monotonic trend with changing excitation density. The effective lifetime is now a time-dependent quantity, , pointing at an out-of-equilibrium process that appears at early times in all measurements. This effect may be considered to be a direct consequence of the presence of interconnected QDs in the samples under analysis. Interconnectivity allows dot-to-dot charge transport and, thus, diffusion of carriers through the QD network to take place. So, when the excitation pulse gives rise to a high charge carrier population (as depicted in Figure 2g), diffusion-mediated redistribution effects lead to a time-dependent reduction of the local average charge carrier density. This redistribution of charge carriers to neighboring QDs causes carriers to access additional defect states in adjacent sites and will thus increase the fraction of trapped carriers, corresponding to the fast initial component observed in TRPL (Figure 2b–d). As fluence increases, more defects will be filled, this process becomes less relevant, and the weight of the fast component is reduced. At the same time, the larger sampling of the defect landscape accounts for the reduced PLQY observed for these *C*_*prec*_, as shown in Figure 1d. These deviations correspond to the initial fast TRPL decay component observed in the TRPL of the connected networks (Figure 2b–d), a signature similar to that reported for some bulk samples^38,39^ and associated with diffusion and fast charge trapping.^40^ In the most extreme case of isolated QDs (Figure 2m), a set of parallel curves are observed, indicating a recombination dynamics that does not depend on the initial excitation density. In this case, all decays are multiexponential, as faster (slower) emitters in an ensemble of QDs will contribute more at early (late) time scales.

### Interconnectivity Determines Emission Efficiency in Perovskite QD Networks

To further understand the impact of the interconnectivity on the macroscopic behavior of the perovskite QD networks under study, we next consider PLQY values as a function of fluence and temperature. This type of measurement allows identification of the prevalent recombination mechanism as a function of the injected carrier density as well as the role played by defect trapping or lattice vibrations. We start the discussion with the bulk scenario, where recombination dynamics depends on the charge carrier population density as described within the SRH model. By extending this model to higher-order recombination mechanisms such as Auger processes, and considering some assumptions,^41^ the recombination dynamics can then be described in a simplified way within the ABC model,^35^ where *A*, *B*, and *C* are nonradiative monomolecular, radiative bimolecular, and nonradiative multiparticle recombination constants, respectively. Assuming only the bimolecular processes to be radiative, the effective emission quantum yield can be calculated as
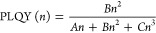
2

**Figure 3:**
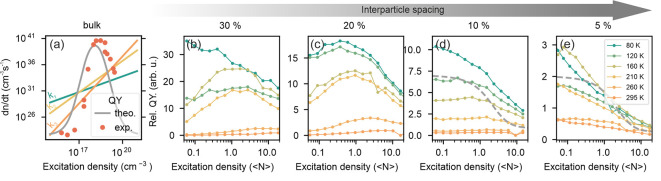
Relative PLQY dependence on excitation density. (a) Expected RT PLQY curve for a FAPbBr_3_ bulk sample (gray line), where excitation-density-dependent values for *A*, *B*, and *C* coefficients (green, yellow, and orange lines) are extracted from TAS measurements. Red dots correspond to the experimental values extracted from a fluence-dependent PL measurement. (b–e) Temperature- and excitation-density-dependent relative PLQY measurements. Dashed gray lines are Poisson distributions indicating the onset of biexciton annihilation in a confined system.

The PLQY fluence dependence of the bulk material (red dots in Figure 3a) follows the theoretically expected bell shape (gray line), estimated considering the recombination coefficients extracted from the fitting of transient absorption spectroscopy (TAS) measurements using the ABC model. These experiments were carried out exploring recombination dynamics over a broad range of carrier densities (see Section S3.1 and Figure S3.1 in the SI). The observed bell shape is indicative of increased dominance of radiative bimolecular recombination due to the filling of charge traps at increasing carrier population.^42^ Above a certain threshold, nonradiative (trimolecular, Auger) recombination processes take over, and the PLQY consequently decreases. Films with higher concentrations of QDs (, , Figure 3b,c) in which interdot connection is more likely, show a similar PLQY bell-shaped curve (for the higher temperatures, this may be more clearly seen in the normalized PLQY curves shown in Figure S3.2). In these examples (see Figure 3b,c) it can be readily observed that, for very low excitation densities, ⟨*N*⟩ ≪ 1, trap-state-assisted recombination gives rise to a largely reduced PLQY, and that for ⟨*N*⟩ ≫ 1, nonradiative multiparticle processes are dominating, also causing a reduction of the PLQY. The optimum PLQY of interconnected QDs is expected at intermediate excitation densities, 1 < ⟨*N*⟩ < 3–5. As the temperature is reduced, two effects can be observed: an overall PL enhancement for each measured fluence and a relative increase in the emission for low fluences. The general PL enhancement is likely the combined result of lower nonradiative phonon-assisted recombination as well as reduced interparticle charge transfer and trapping. On the other hand, the higher relative rise in PL taking place for low fluences indicates a passivation of thermally activated defect states or a reduction in the trapping rate *k*_*trap*_. In the regime where recombination is trap-assisted (⟨*N*⟩ = 0.06, i.e., the ratio between the density of photons absorbed, calculated from the fluence employed and the absorptance, and the density of QDs in the film, estimated as explained in the Supporting Information, Section S1), an emission enhancement of almost 4 orders of magnitude is observed as the temperature is reduced, while emission dominated by multiparticle processes (⟨*N*⟩ = 48) only rises by 1 order of magnitude (see Figure S3.3), indicating that deactivation of trapping pathways is a key factor in the emission properties.

If we now consider the system with larger average separation between QDs (), where interconnectivity should be absent, a drastic change in the PLQY is observed (Figure 3e). First, the overall PL enhancement as *T* is reduced is now much smaller (3-fold factor) than the one observed for the high *C*_*prec*_ samples (3–4 orders of magnitude), very likely because temperature-induced changes in charge transport are now absent, given the larger interparticle distance. The absence of dot-to-dot transport implies that the likelihood of a charge carrier getting trapped depends exclusively on the probability of a QD possessing a trap state, which is independent of the excitation density. Consequently, the monotonically growing initial part of the bell-shaped PLQY curve is lost. This effect also explains the much smaller PLQY observed for the films with shorter *d*_*QD*_ at low fluences (see Figure 1b). As the preparation methods of the different samples under analysis only differ in the concentration of precursors employed, we can assume that the defect density per QD is very similar in the *C*_*prec*_ = 30% and 5%, the potentially small variations between them not being enough to justify such large differences in PLQY. However, as the spacing between QDs is larger, the defect landscape that carriers can access is reduced, hence increasing the chances of decaying through a radiative path. This finding can be generalized to any assembly of interconnected QDs: recombination in the low-fluence regime is not only determined by the overall defect density in the material system but also by the presence of charge transport, which allows carriers to sample a wider defect landscape. As for the high-fluence regime (⟨*N*⟩ > 1 for highly confined systems), two-particle biexciton annihilation is responsible for the drop of PLQY observed.^43^ Interestingly, the results attained for the  and  samples follows the Poisson function that provides the probability of exciting the same QD at a given average excitation density (dashed line in Figure 3d,e), assuming any excitation above 1 leads to nonradiative biexciton annihilation.^44^ In contrast, higher *C*_*prec*_ samples show a multiparticle onset at higher excitation densities, which corresponds to Poisson distributions with a larger probability for radiative recombination allowed per QD, as evidenced in Figure S3.4. This is consistent with a less efficient biexciton annihilation caused both by an increasing QD size (see Table 1) and, most importantly, the loss of confinement induced by the transport of charges between neighboring dots, in good agreement with all previous observations.

### PL Peak Broadening in QD Networks: Interplay of Connectivity and Nanocrystal Size Polydispersity

**Figure 4:**
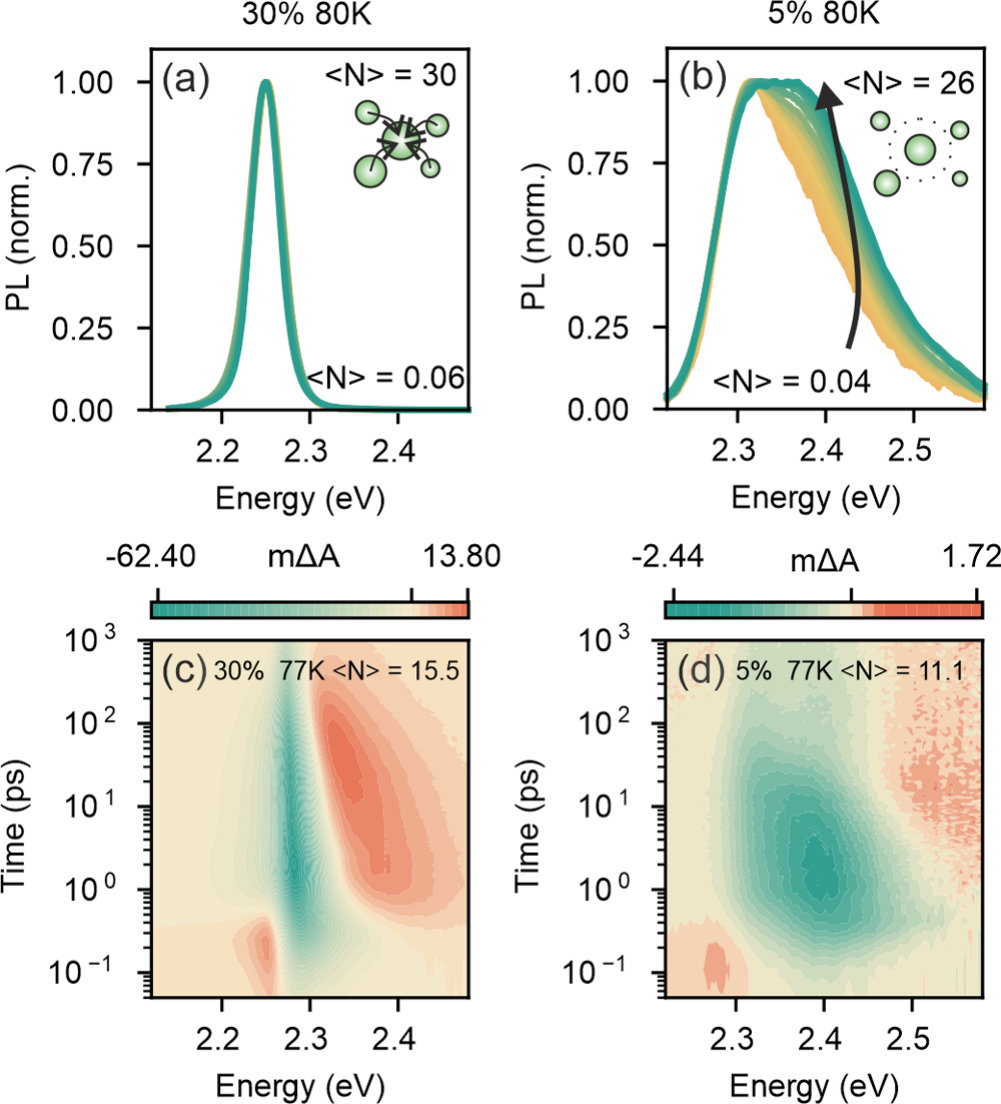
Low temperature spectral shape of PL and TAS features. (a,b) Fluence-dependent PL measurements at 80 K for (a) 30% and (b) 5% *C*_*prec*_ samples. Insets illustrate the observed charge carrier funneling process. (c,d) High-fluence TAS spectra represented in color maps for (c) 30% and (d) 5% *C*_*prec*_ samples.

As stated in previous sections, the higher or lower level of connectivity between neighboring QDs affects the defect landscape to which carriers have access. Hence, connectivity becomes, along with size polydispersity and electron–phonon coupling,^45,46^ a potential source of spectral broadening of the photoemission. While an exact estimation of the size distribution in our samples is not trivial, we expect the size dispersion to be very similar for all *C*_*prec*_ employed. Thus, in order to estimate the role of connectivity in the emission of our QD networks, we performed low-temperature measurements where electron–phonon coupling is largely reduced. With this in mind, we realized a comparative analysis of the PL emission spectra attained at 80 K of the samples that display the largest difference in average interdot separation, namely, *d*_*QD*_ = 12 nm and *d*_*QD*_ = 28 nm. Results, shown in Figure 4, evidence the reduction of electron–phonon coupling contribution, since strong variations in the spectral width of the PL, *w*_*PL*_, are observed that are not present in RT measurements (see Figure S4.1).

Further, strong differences in both *w*_*PL*_ and its evolution with fluence are observed when the QD connectivity is modified. If we consider the sample for which interdot separation is expected to be shortest (), the PL is spectrally narrow and does not vary with excitation fluence (Figure 4a). This behavior can be understood as the result of the funneling of photogenerated charges toward large QDs, which act as recombination centers and thus as the direct consequence of dot-to-dot transport. If we turn to the sample containing isolated QDs (), a different behavior is observed (Figure 4b). On the one hand, an overall spectrally broader PL is evident. This is to be expected as we are now measuring the additive PL from a collection of noninteracting QDs. On the other hand, a broader PL is measured as we increase the pump fluence. This fact can be explained as the consequence of the size-dependent absorption cross section of the different QDs (see the discussion in Section S4.1).

The hypothesis of interdot connectivity favoring carrier transfer is further supported by independent ultrafast TAS measurements (see Figure 4c,d). The time evolution of the TAS spectra of the highly connected QD sample (*d*_*QD*_ = 12 nm) presents a fast (1 ps) initial narrowing of the ground state bleach (GSB) associated with the cooling of photoexcited hot-carriers in the sample (Figure 4c). As the interparticle spacing is increased, the GSB feature becomes broader and presents a spectral narrowing (for energies around 2.5 eV) taking place over much longer times, up to 10 ps for the case of separated QDs (*d*_*QD*_ = 28 nm) (Figure 4d). The broader GSB feature is again the result of inhomogeneous broadening caused by the presence of a dispersion of isolated QDs and the longer times over which spectral narrowing takes place are similar to those reported in the past for cooling of hot-carriers in isolated lead halide perovskite colloidal nanocrystals.^47^ A proper description of cooling dynamics in these systems certainly demands a more detailed study, which is out of the scope of the present work.

### Multiparticle Recombination Effects in Localized and Interconnected Emitters

**Figure 5:**
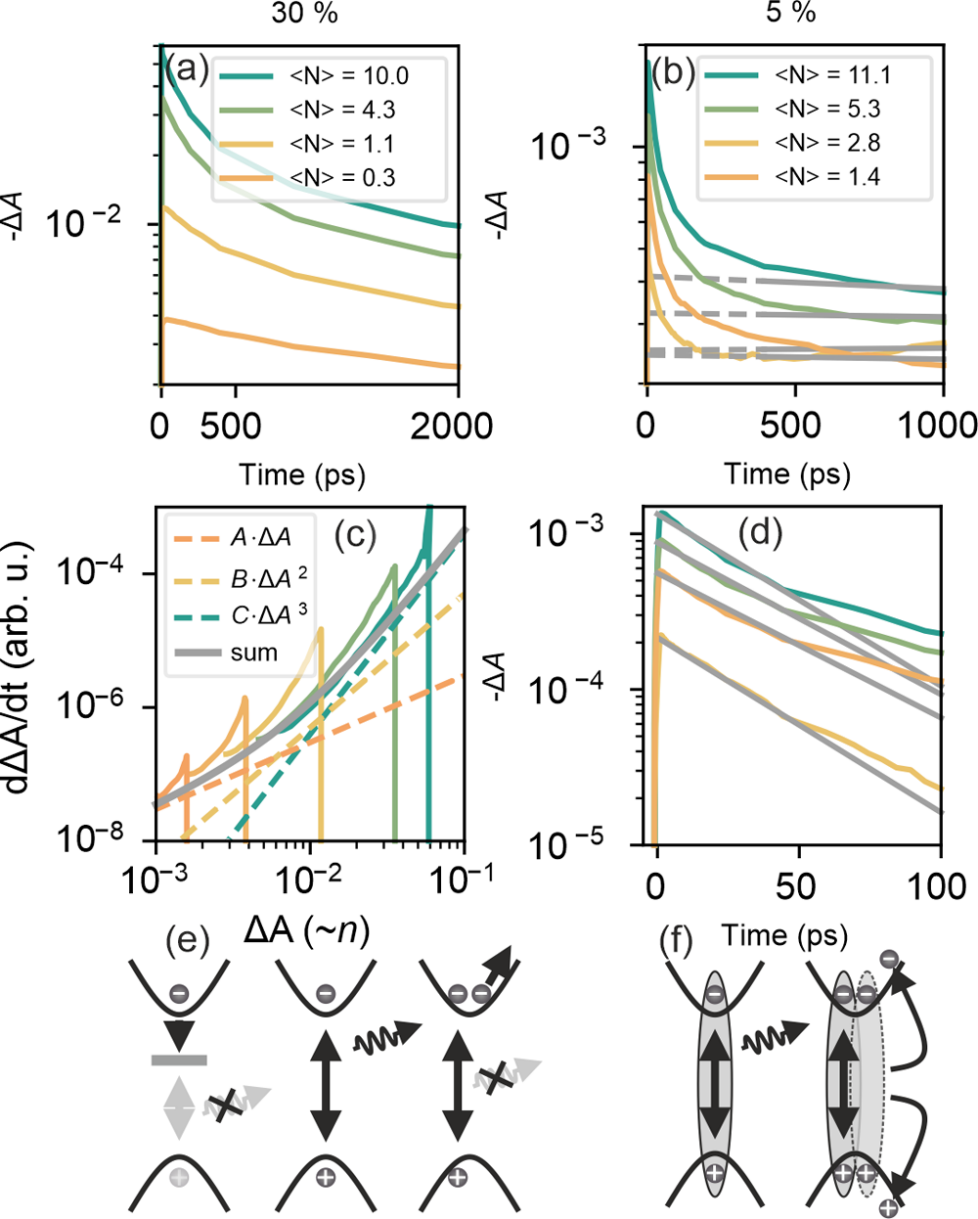
Ground state bleach dynamics in samples with different degrees of QD connectivity. (a,b) GSB decays of (a) *C*_*prec*_ = 30% and (b) *C*_*prec*_ = 5% samples extracted as the minimum from the TAS signal. Gray dashed lines in (b) indicate the Δ*A* background level mentioned in the text. (c) Derivative representation of the GSB data shown in (a). Gray line indicates sum of graphically approximated *A*, *B*, and *C* decay rates (green, yellow, and orange dashed lines, respectively). (d) Difference of the GSB decay and the Δ*A* background indicated in (b). Gray lines represent single exponential fits (fit values in SI). (e) Sketch of the proposed dominating ABC model for the *C*_*prec*_ = 30% sample. (f) Sketch of the biexciton annihilation process associated with the dynamics of the *C*_*prec*_ = 5% sample.

In this section, we further explore the regime where a high carrier density is injected into the QD solid. This scenario, of relevance for lasing applications, is where nonradiative multiparticle processes can dominate recombination representing one of the main burdens for achieving optical gain.^3^ To explore the role of QD connectivity in this regime, we have performed fluence-dependent TAS measurements and studied the time dynamics of the GSB extracted as the minimum *ΔA* value at each time instant (Figure 5a,b). In a bulk-like system, the above-described ABC model has been broadly employed to describe recombination as a function of the density of photogenerated carriers.^48^ While good fits were obtained for the reference bulk sample presented before (see Figure S3.1), deviations from the model become evident when we consider QD arrays. For the highly interconnected *d*_*QD*_ = 12 nm sample, deviations of the experimental data from the model can be clearly seen when plotting the time derivative of the *ΔA* against *ΔA* (Figure 5c). In analogy to the TRPL data shown above, this representation allows discrimination between time-dependent (τ(*n*,*t*)) and time-independent contributions (τ(*n*)). Under equilibrium conditions, where charge carriers are homogeneously distributed both spatially and energetically, the *ΔA* at the GSB is expected to be proportional to the charge carrier density.^49^ In this scenario, a given charge carrier density should lead to a certain decay rate and, consequently, a fixed value for its time derivative. Changing the initial carrier density in a TAS experiment would lead to overlapping dynamics in a  vs *ΔA* plot. For the *d*_*QD*_ = 12 nm sample, we can observe early stage deviations which can be associated with charge carrier energy homogenization processes that comprise carrier diffusion as well as thermalization, the latter being strongly affected by confinement and Auger processes.^50^ After ∼2 ns, the TA line shape does not show any significant change even at elevated excitation densities (compare Figure S4.2), and the GSB decays overlap in their final stages, when carriers are assumed to be in equilibrium, and can be simulated with the ABC model as indicated by the gray line in Figure 5c.

In contrast, small and isolated QDs, *d*_*QD*_ = 28 nm, show a clearly distinct behavior, where the GSB decay can be separated into a fast initial component followed by slow decay into a *ΔA* background level that increases only slightly as expected for a distribution of populated QDs that follows Poisson statistics (Figure 5b). This behavior is characteristic of quantum confined nanostructures presenting excitonic recombination where the fast initial trace can be associated with the biexciton annihilation and the slower background with exciton recombination.^43,44^ If the *ΔA* background level is subtracted from the general decay, the resulting signal can be fitted to a single exponential (Figure 5d) that yields a fluence-independent decay rate of 42 ps associated with the biexciton lifetime. This value is a factor of 2 smaller than previously reported for colloidal FAPbBr_3_ NCs.^51^ This small discrepancy is likely a combination of several factors such as the larger NC size used in ref (51) (10.5 nm) as well as the absence of any ligands in our samples.

## Conclusions

In summary, we have unveiled the effect of dot interconnectivity on the charge carrier transport and recombination occurring in perovskite QD networks. In order to do so, we have taken advantage of a series of ligand-free QD assemblies with different degrees of connectivity (including isolated particles) attained by loading a nanoporous film with varying QD filling fractions. Employing different time-resolved spectroscopic techniques, we have followed the carrier dynamics over a broad time and fluence range, which comprises carrier density regimes relevant for both photovoltaic as well as light-emitting applications. While samples presenting a low QD concentration can be described in the framework of isolated nanostructures, increasing the load leads to enhanced connectivity and a scenario where charge diffusion dictates the optoelectronic response. We find that charge diffusion, leading to a spatial homogenization within the QD solid, allows for charge transport and enhances the defect landscape accessible to each carrier. Our results imply that a judicious tuning of the connectivity among QDs should lead to a scenario where charge transport, needed for most optoelectronic applications, may be optimized by considering the balance between the demand for carrier density and the effect it has on the available recombination paths. Also, the analysis and concepts herein discussed may be extended and applied to QD solids of different morphology and composition, as they will allow individuation of the different recombination mechanisms for photoexcited carriers and contribute to a precise device-oriented design.

## Methods

### Sample Fabrication

#### Materials

Formamidinium bromide (FABr, GreatCell Solar Materials, 99.9%), lead(II) bromide (PbBr_2_, TCI, 99.99%), dimethyl sulfoxide (DMSO, *Merck*, anhydrous 99.8%), methanol (MeOH, *VWR*, 98%), ethyl acetate (EA, VWR, anhydrous), and chlorobenzene (CB, *Merck*, 99.9%) were used without additional purification steps.

#### Preparation of SiO_2_ Nanoparticle Porous Scaffold

Commercially available 30 nm SiO_2_ nanoparticles (34% w/v in H_2_O, LUDOX-TMA, Sigma-Aldrich) were diluted in methanol to 3% w/v to obtain a colloidal suspension. The diluted suspension was deposited on top of a low-fluorescence glass substrate via dip coating, employing a withdrawal speed of 120 mm/min. To achieve a thickness of around 1 μm, the deposition was repeated 15 times and consequently annealed at 450 °C for 30 min to remove any residual organic component in the matrix and to improve its mechanical stability.

#### Synthesis of FAPbBr_3_ QDs within Nanoporous Silica Scaffold

A perovskite solution precursor was prepared using FABr and PbBr_2_ powders dissolved in DMSO in a 1:1 molar ratio at different concentrations. By spin-coating (5000 rpm for 60 s), the solution was infiltrated in the void spaces of the scaffold, followed by a heat treatment at 100 °C for 1 h to obtain FAPbBr_3_ QDs within the pores of the matrix. The synthesis was conducted in a protected N_2_ environment in a glovebox. As a reference, a FAPbBr_3_ BULK film was prepared using a perovskite precursor solution of 30% wt concentration spin-coated on top a low-fluorescence glass using a two-step process (500 rpm, 5s; 3000 rpm, 90s). 200 uL of EA are added dropwise after 30s during the second step. A final annealing at 100 °C for 1 h gives rise to perovskite crystallization.

#### Electron Microscopy

TEM images were acquired using an FEI Talos F200S scanning/transmission electron microscope operated at 200 kV from lamellae obtained with a focused ion beam (FIB, Carl Zeiss, Auriga).

### Optical Characterization

#### Absolute Photoluminescence Quantum Yield Measurements

PLQY was measured in a commercial fluorometer (*Edinburgh* FLS1000) using an excitation wavelength of λ = 450 nm with the sample contained in an integrating sphere accessory.

#### Time-Resolved Photoluminescence Measurements

Time-resolved measurements were carried out using a 200 kHz pulsed white light source (*NKT Fianium SuperK*) filtered by an acousto-optic tunable filter to an excitation bandwidth ranging from 450 to 470 nm in combination with an *id100* single photon detector (*ID Quantique*) and a *SPC-130-EMN* time correlated single photon counting detection card (*Becker and Hickl*). For precise fluence estimation, the spot size was controlled with an *Ophir SP932U* beam profiling camera, and power was continuously measured with a beam sampling configuration.

#### Relative Photoluminescence Quantum Yield Measurements

Temperature-dependent relative PLQY measurements were performed using a home-built setup employing a pulsed (1 kHz) ultrafast laser (λ_c_ = 1030 nm) *Pharos* coupled to an *Orpheus* collinear optical parametric amplifier (both from *Light conversion*) set to emit ∼200 fs, 450 nm excitation pulses. The power and beam profile were continuously measured with a beam sampling configuration and adapted with automated filter wheels. The sample was placed in an *OptistatDN* cryostat (*Oxford instruments*), and detection is carried out with a *Flame* spectrometer (*Ocean Insight*), where the acquisition time was adapted to increase the dynamic detection range and detection counts where normalized to the acquisition time. All hardware components and data acquisition were fully automated and controlled by a self-written *Python* program.

#### Transient Absorption Spectroscopy Measurements

Transient absorption measurements were performed using a commercial transient absorption spectroscopy setup (*HELIOS* Femtosecond Transient Absorption Spectrometer from *Ultrafast Systems*) using the same excitation as with the relative photoluminescence QY measurements. Cryogenic measurements where performed employing the above-mentioned cryostat.

## Supporting Information Available

Size and interparticle distance estimation; Shockley–Read–Hall charge carrier recombination model and fit results; Distribution of effective lifetimes in isolated QDs; Details on photon flux representation of TRPL data; Temperature- and fluence-dependent PLQY data: ABC model for FAPbBr_3_, normalized excitation density and temperature-dependent PLQY, temperature-dependent PL enhancement, simulation of Poisson distributed excitation scenarios; Size distribution and charge carrier funneling effects: Spectral analysis at different temperatures and fluences, energy-resolved state filling dynamics evidenced by TAS (PDF)

## Notes

*Preprint version*. A preprint version of this work can be found at: David O. Tiede, Carlos Romero-Pérez, Katherine A. Koch, K. Burak Ucer, Mauricio E. Calvo, Ajay Ram Srimath Kandada, Juan F. Galisteo-López, Hernán Míguez. Effect of connectivity on the carrier transport and recombination dynamics of perovskite quantum dot networks. *arXiv*, 2023, 2310.12719. https://arxiv.org/abs/2310.12719 (accessed October 19, 2023).
