# Supplementary material for: Effect of Connectivity on the Carrier Transport and Recombination Dynamics of Perovskite Quantum-Dot Networks
Source: ACS Nano. 2024 Jan 11;18(3):2325–34. doi: 10.1021/acsnano.3c10239 (PMC10811662; doi:10.1021/acsnano.3c10239)
Supplement: Supplementary file 1 — nn3c10239_si_001.pdf [file nn3c10239_si_001.pdf]

Supporting Information for:

Effect of connectivity on the carrier transport and recombination dynamics of perovskite quantum dot networks

David O. Tiede[1], Carlos Romero-Pérez[1], Katherine A. Koch[2], K. Burak Ucer[2], Mauricio E. Calvo[1], Ajay Ram Srimath Kandada[2], Juan F. Galisteo-López[1]*, Hernán Míguez[1]*.

[1]Instituto de Ciencias de Materiales de Sevilla (Consejo Superior de Investigaciones Científicas-Universidad de Sevilla), C/Américo Vespucio, 49, Sevilla, 41092, Spain.

[2] Department of Physics and Center for Functional Materials, Wake Forest University, 1834 Wake Forest Road, Winston-Salem, NC 27109, USA.

**E-mail: juan.galisteo@csic.es, h.miguez@csic.es*

Contents

S1 Structural characterization 2

S1. 1 Size and interparticle distance estimation from fill fraction and optical measurements 2

S2 Trap filling dynamics in isolated and interacting QD networks 3

S2.1 Shockley – Read – Hall charge carrier recombination model. 3

S2.2 Distribution of effective lifetimes in isolated QDs 4

S2.3 Photon flux representation of TRPL data 5

S3 Temperature and fluence dependent PLQY data 7

S3.1 ABC model for bulk FAPbBr3 7

S3.2 Normalized excitation density and temperature dependent PLQY 8

S3.3 Temperature dependent PL enhancement 8

S3.4 Simulation of Poisson distributed excitation scenarios 9

S4 Size distribution and charge carrier funneling effects 10

S4.1 Spectral analysis at different temperatures and fluences 10

S4.2 Energy resolved state filling dynamics evidenced by TAS 11

References 11

S1 Structural characterization

S1. 1 Size and interparticle distance estimation from fill fraction and optical measurements

Average perovskite quantum dot (QD) sizes for the different samples under study were estimated by using the Brus equation, [1] an approach that has proven to yield a good estimate in the past for similar samples: [2]

$$E_{g,nano} = E_{g,bulk} + \frac{h^2}{8\mu R^2} - \frac{1.786e^2}{4\pi\varepsilon_0\varepsilon_r R} \qquad (1)$$

where the values for effective mass (μ) and dielectric constant (ε_r) were taken from reference [3] and the bandgap estimated from PL measurements of the bulk sample. This equation assumes a fixed set of parameters for effective mass of electron m_e^*, hole m_h^* as well as for the dielectric constant and assumes this set to remain unchanged under confinement. Further, the estimation of the bulk bandgap from the emission energy contains uncertainties since the effect of an excitonic transition is not considered. In addition, possible effects from size distribution and charge funneling effects and modifications in the electronic band structure through e.g. strain are not reflected in eq. 1. Despite the uncertainties introduced by the mentioned effects, we employ the Brus equation to get an estimation of particle sizes to obtain a figure of comparison between the different samples rather than to obtain an exact value for the actual size and size distribution. The estimated average sizes are shown in **Table S1**.

Table S1 Fill fraction, estimated average QD radius and interparticle spacing extracted from porosimetry and optical measurements.

Precursor concentration C_{prec}	Fill fraction *ff*	Estimated <*r*>	# of QDs per m^3	d_{center}	d_{interp}
5 %	0.0141	4.5 nm	$3.7 \cdot 10^{22} m^{-3}$	37 nm	28 nm
10 %	0.0405	5.7 nm	$5.2 \cdot 10^{22} m^{-3}$	33 nm	22 nm
20 %	0.092	6.26 nm	$9.0 \cdot 10^{22} m^{-3}$	28 nm	15 nm
30 %	0.145	6.67 nm	$8.0 \cdot 10^{23} m^{-3}$	25 nm	12 nm

To estimate the interparticle spacing between QDs, we assume the particles to be equally spaced within the host volume. In that case, in a volume V filled with N particles, the average volume v per particle is given by

$$v = \frac{V}{N} \qquad (2)$$

Where, assuming spherical particles, the volume can be expressed as

$$v = \frac{4}{3}\pi r^3 = \frac{V}{N} \qquad (3)$$

To obtain the average distance between particle centers, the previous equation can be rewritten as:

$$d_{center} = 2r = 2\sqrt[3]{\frac{3V}{4\pi N}} \qquad (4)$$

The average void distance between particles can consequently be estimated as

$$d_{interp} = d - 2\langle r\rangle \qquad (5)$$

From this estimation, the values shown in **Table 1** can be extracted. It becomes evident that the average particle size is only changing by a factor of 1.5, while the interparticle spacing is more than doubled.

S2 Trap filling dynamics in isolated and interacting QD networks

S2.1 Shockley – Read – Hall charge carrier recombination model.

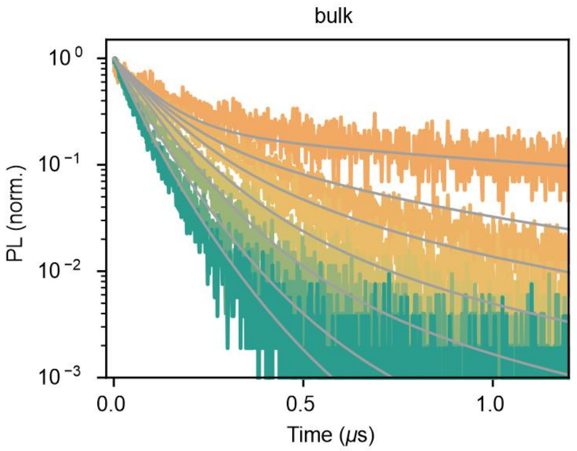

Figure S2.1 Shockley- Read-Hall model for bulk $FAPbBr_3$. Normalized fluence dependent TRPL data with fluences ranging from $8.9 \cdot 10^{14} cm^{-3}$ (orange curve) to $1.7 \cdot 10^{17} cm^{-3}$ (green curve). Grey lines represent global fit to the SRH model described below.

The Shockley -Read – Hall (SRH) model describes the charge carrier recombination dynamics with a set of differential equations for the change in the population density of electrons n_e, trapped electrons n_T and holes n_h. [4] Given the low excitation regime, we exclude Auger effects from the model and assume the following rate equations:

$$\frac{dn_e}{dt} = -R_{pop}(N_T - n_T)n_e + R_{detrap}n_T - R_{eh}n_e n_h \quad (6)$$

$$\frac{dn_T}{dt} = +R_{pop}(N_T - n_T)n_e - R_{detrap}n_T - R_{depop}n_T n_h \quad (7)$$

$$\frac{dn_h}{dt} = -R_{depop}n_T n_h - R_{eh}n_e n_h \quad (8)$$

, where R_{pop}, R_{detrap}, R_{eh}, R_{depop} describe the electron trapping rate, the detrapping rate of an electron returning to the conduction band, the radiative electron-hole recombination rate and the non-radiative trapped electron-hole depopulation rate, respectively. Note that we do not discriminate between possible electron or hole defects and that the same result could be reproduced with hole traps instead of electron traps. The global fit of the experimental time-resolved photoluminescence (TRPL) data results in the following rate constants:

$$R_{pop} = 3.39 \cdot 10^{-8} \mathrm{cm^3 s^{-1}}$$

$$R_{detrap} = 5.74 \cdot 10^{7}\ \mathrm{s^{-1}}$$

$$R_{depop} = 2.99 \cdot 10^{-9} \mathrm{cm^3 s^{-1}}$$

$$R_{eh} = 3.60 \cdot 10^{-11} cm^3 s^{-1}$$

$$N_T = 1.80 \cdot 10^{15} cm^{-3}$$

S2.2 Distribution of effective lifetimes in isolated QDs

Isolated QD samples show a distinct behavior as compared to interconnected ones both in static and dynamic PL measurements. The PL spectrum for the C_{prec}=5% sample (**Fig. S2.2a**) is broader than the rest of the samples under consideration where a certain degree of interconnection is expected. Further, TRPL measurements show a spectral dependence, which is expected for a sample comprising a distribution of independent QDs. The PL decay when only the high energy components of the PL spectrum are considered (green band in **Fig. S2.2a**) is much faster than for the low energy components (orange band), which match the overall integrated decay (**Fig. S2.2b**). The PL decays show a multi-exponential behavior that could be best fitted to a lognormal distribution of decay rates (**Fig. S2.2c-e**).[5] From these fits a lifetime distribution can be retrieved for each spectral range (**Fig. S2.2f-h**) where it becomes evident the presence of a distribution of isolated QDs with different emitting properties. Here it should be mentioned that the presence of QDs with faster PL decay and higher energetic emission cannot be exclusively associated with the QD size but other factors must be considered such as a varying density of defects, modifications in the energy levels due to a modified crystal geometry or the presence of strain.

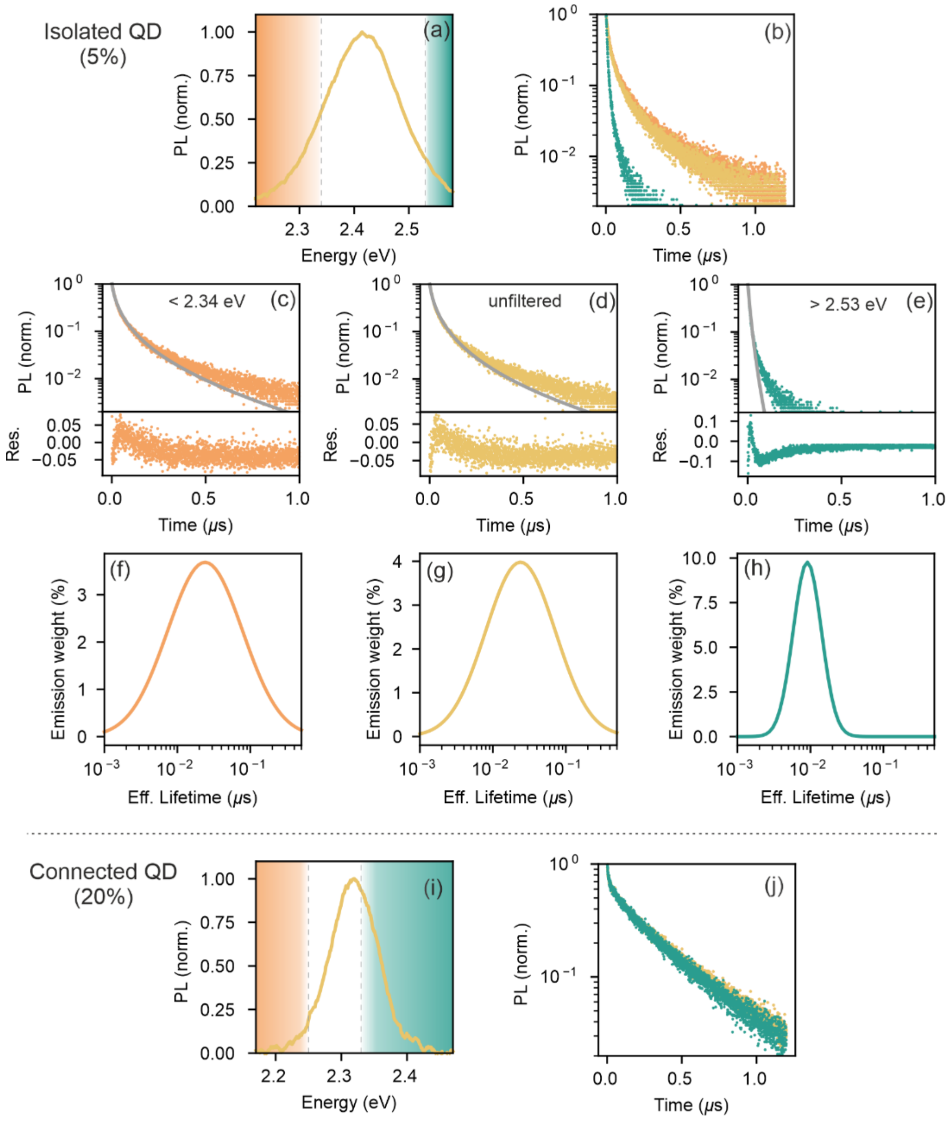

Figure S2.2 Spectrally resolved effective lifetime distributions of isolated QDs (C_{prec} = 5%). (a) PL emission spectrum of C_{prec} = 5% at RT. Shaded green (orange) area indicates high (low) energetic emission ranges

considered in the following panels. (b) Comparison of TRPL decays detected without spectral detection filter (yellow dots), detected with energies below 2.34eV (orange dots) and above 2.53eV (green dots). Color code used for the different detection ranges is identical for all panels. (c)-(e) Fit (grey lines) of TRPL decays to a log normal distribution of effective lifetimes. Bottom panel shows residual values of fit. (f)-(h)Emission weight of the effective lifetimes extracted from (c)-(e). (i)-(j) PL emission spectrum of $C_{prec} = 20\%$ at RT. (j) Corresponding TRPL decays (excitation density $0.4 < N >$) from different spectral regions as indicated by color coding in (i).

The mean effective lifetime of the high energetic emission is ~ 10 ns= $1 \cdot 10^{-8}s$. This corresponds to the fastest possible emitter, where no emission delaying through defect trapping and detrapping or other delaying effects such as polaron formation or modification of the electronic band structure through strain take place. In the SRH model, the direct electron-hole recombination $R_{eh} = 3.6 \cdot 10^{-11} cm^3 s^{-1}$ can be used to estimate the radiative recombination rate in a defect free QD, assuming $V_{NC} = 3.8 \cdot 10^{-25} m^3$, two charges per excitation and $\tau^{-1} = k\rho = R_{eh} \cdot \frac{2}{V_{Nc}} = (5.3 \cdot 10^{-11} s)^{-1}$ $(1.6 \cdot 10^{-10} s$ for $C_{prec} = 30\%$. This effective lifetime extracted from the population density dependent SRH model can be further slowed down through retardation effects such as thermalization, exciton formation, polaron formation or (surface-) strain induced deformation of the electronic band structure, such that the mismatch between the SRH value and the value extracted from the log-normal distribution can be explained. Nevertheless, this simplified calculation demonstrates that pristine QD are likely to show a strongly accelerated recombination dynamic and that a reduction in V_{NC} leads to an acceleration in recombination time.

S2.3 Photon flux representation of TRPL data

TRPL data from the different samples under consideration (shown if **Fig.2a-e** of the main text) were also represented as photon flux vs. its time derivative in order to identify out-of-equilibrium processes taking place during charge carrier recombination. In order to perform such representation (**Fig.2i-m**) TRPL data were approximated with a multiexponential decay function, where the number of exponential decay terms was varied in order to reproduce the experimental data as close as possible. The multiexponential decay function was derived analytically and plotted against the amplitude of the PL flux for each data point.

Charge carrier recombination models mentioned in the main text (SRH and ABC models) describe the recombination of excited carriers in an equilibrium situation where they are homogenously distributed in space within a semiconductor and lying at the maximum (minimum) of the valence (conduction) band. Under this assumption, the time derivative of the photon flux should follow a monotonous trend where the slope will vary reflecting which process dominates recombination (monomolecular, bimolecular or higher order processes) for a given carrier density. If different measurements are performed for varying initial injected carrier densities n_0, experimental data should fall along the mentioned monotonous trend.

Any out-of-equilibrium process will then represent a deviation from this expected trend. These situations can imply energetic deviations (carriers are not lying at band extrema as in the case of a population of hot carriers) or carriers are undergoing spatial diffusion before reaching a homogeneous spatial distribution within the matrix. With the time resolution of our experiments we are probing a population of thermalized carriers thus any deviation from the expected trend should come from carrier diffusion processes. Under these circumstances, when plotting the data for several measurements with varying n_0, an out-of-equilibrium situation should appear in the initial stages where a spatially homogeneous distribution of carriers has not been achieved. Such deviations are absent in the bulk and non-connected QD (C_{prec}=5%) samples indicating that charge

diffusion is either too fast (bulk) or not present (separated QDs). For intermediate samples an out of equilibrium process becomes evident at the beginning of each measurement showing the presence of diffusion. The reason why diffusion is taking such long times as to become evident in this representation is due to two (connected factors): charges must be transferred from one QD to the adjacent one and in this process, they have access to a larger defect landscape (as they are able to probe traps states present at neighboring QDs).

The longer, converged tails of the decay curves can be described within an equilibrium situation, where trap filling ($R_{pop}(N_T - n_T)n_e$) and detrapping ($R_{detrap}n_T$) have equilibrated. In this scenario, eq.6 and eq.7 simplify to:

$$\frac{dn_e}{dt} = -R_{eh}n_e n_h \qquad (9)$$

$$\frac{dn_h}{dt} = -R_{depop}n_T n_h \qquad (10)$$

Further, the trapped carrier density can be expressed as a function of electron density as:

$$\frac{dn_T}{dt} = +R_{pop}(N_T - n_T)n_e - R_{detrap}n_T - \underbrace{R_{depop}n_T n_h}_{\ll R_{pop}} \qquad (11)$$

$$n_T = \frac{R_{pop}N_T n_e - \frac{dn_T}{dt}}{(R_{detrap} + R_{depop}n_h + R_{pop}n_e)} \approx \frac{R_{pop}N_T n_e}{(R_{detrap} + R_{pop}n_e)} \qquad (12)$$

, as $R_{depop}n_T n_h, \frac{dn_T}{dt} \ll R_{pop}N_T n_e$. Next, due to charge neutrality, the hole density can be expressed as a function of electron and trapped electron density:

$$n_h = n_e + n_T = n_e + \frac{R_{pop}N_T n_e}{(R_{detrap} + R_{pop}n_e)} \qquad (13)$$

The y-axis in the photon flux representation is given by the derivate of the PL signal, which can be now written as:

$$\frac{dPL}{dt} = R_{eh}\frac{dn_e n_h}{dt} = R_{eh}\frac{dn_e}{dt}n_h + R_{eh}n_e\frac{dn_h}{dt}$$

$$= R_{eh}\big(R_{eh}n_e n_h \cdot (n_e + n_h) + R_{depop}n_T n_h \cdot n_e\big) \qquad (14)$$

The x-axis of the photon flux representation is given by the amplitude of the TRPL signal, which is proportional to the densities of electrons and holes ($PL \propto R_{eh}n_e n_h \propto n_e n_h$). Now, two scenarios can be considered:

- Trap recombination dominated, monomolecular regime ($n_h, n_T \gg n_e$):
  Here, the majority of electrons get trapped, such that $n_T \approx n_h$. Since $R_{depop} > R_{eh}$, **eq. 14** simplifies to:

  $$\frac{dPL}{dt} \propto R_{depop}n_T n_h n_e \qquad (15)$$

  In this regime, the recombination dynamics are effectively dominated by two charge carrier densities, such that a representation of the product of two charge carrier densities against the product of two charge carrier densities results in a slope of $m = 1$ in a log-log representation (**see Fig. S2.3**).
- Electron-hole recombination dominated, bimolecular regime ($n_e \approx n_h$):

Here, the number of free electrons exceeds the number of trapped electrons ($n_e > n_T$). In this case, in **eq. 14** the electron-hole recombination dominates, such that

$$\frac{dPL}{dt} \propto R_{eh} n_e n_h \cdot (n_e + n_h) \qquad (16)$$

In this regime, the recombination dynamics are dominated by the product of three charge carrier densities. A representation of the product of three charge carrier densities against the product of two charge carrier densities results in a slope of m =1.5 in a log-log representation (**see Fig. S2.3**).

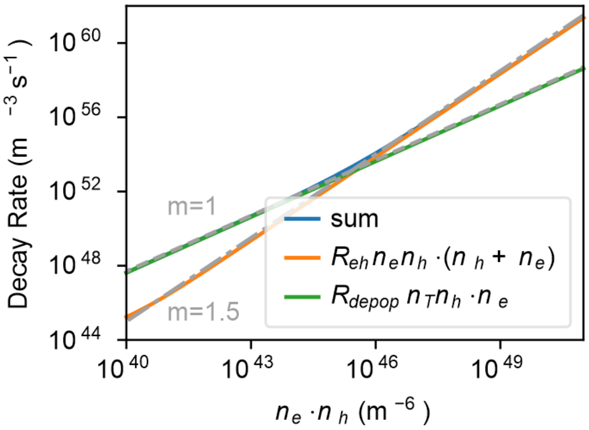

Figure S2.3 Simulation of the decay rates of electron-hole and trap depopulation contributions. At low charge carrier densities, the trap depopulation mechanism with a slope of $m = 1$ dominates. The higher charge carrier density regime is dominated by the electron hole recombination that has a slope of $m = 1.5$. The curves are simulated with the fit values of section S2.1.

S3 Temperature and fluence dependent PLQY data

S3.1 ABC model for bulk $FAPbBr_3$

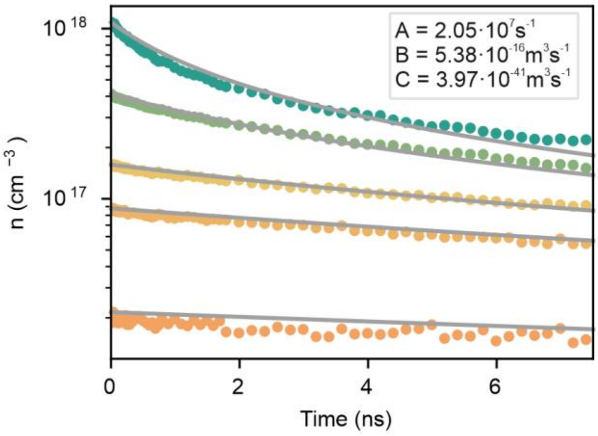

Figure S3.1 GSB decay dynamics of FAPbBr3 bulk. Fluence dependent GSB dynamics fitted globally to the ABC model as described in the main text.

Room temperature fluence dependent transient absorption spectroscopy (TAS) was performed on $FAPbBr_3$ bulk samples and the GSB taken as the minimum value of the TAS spectra. The ΔA GSB signal was converted into charge carrier density by establishing a relationship between the injected charge carrier density and initial GSB signal for each fluence. The time evolution of the GSB was adjusted to the ABC model:

$$\frac{dn}{dt} = -A \cdot n - B \cdot n^2 - C \cdot n^3 \qquad (17)$$

Excellent fits were obtained (see **Fig. S3.1**) from which values for the three recombination rates were obtained: $A = 2.05 \cdot 10^7 s^{-1}$, $B = 5.38 \cdot 10^{-16} m^3 s^{-1}$ and $C = 3.97 \cdot 10^{-41} m^6 s^{-1}$.

S3.2 Normalized excitation density and temperature dependent PLQY

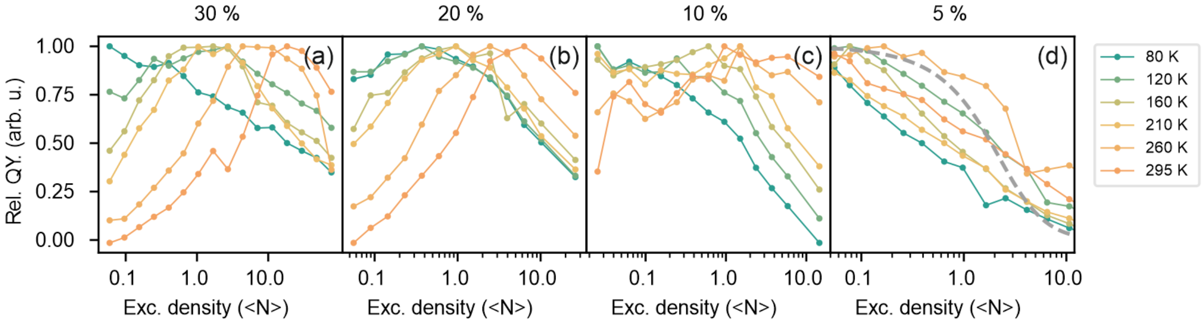

Figure S3.2 Normalized PLQY data for different QD samples. (a) – (d) PLQY curves normalized to their maximum PLQY for different C_{prec} values. Dotted line in (d) indicates normalized PLQY expected for a Poisson distribution of excitations per QD where only one recombination per QD is radiative.

S3.3 Temperature dependent PL enhancement

As discussed in the main text, the temperature dependence of the PL in the NC solids under study is strongly influenced by the carrier density which determines which recombination regime dominates. Here we discuss two extreme cases. For the highly interconnected samples with $C_{prec} = 30\%$ (**Figure S3.3a**) emission undergoes a drastic enhancement of 4 orders of magnitude as T is reduced from RT to 77K in the low fluence scenario, where charge carrier recombination is dominated by trap-assisted processes. This is, as discussed, likely a combination of passivation of thermally activated defect states and a reduction in the trapping rate k_{trap}. For higher fluences the PL enhancement is only slightly above an order of magnitude.

For the isolated case ($C_{prec} = 5\%$) (**Figure S3.3b**), enhancement across all fluences is much weaker (below an order of magnitude) since the role of defect assisted recombination is reduced as carriers cannot access defect traps in neighboring QDs. In addition, this sample shows characteristic PLQY enhancement around phase transition temperatures. [6] Such enhancement becomes more evident for isolated QDs as charge funneling processes, that can hide out local variations induced by the phase transition of individual QDs, are prevented. A detailed discussion of those dynamics is beyond the scope of this work.

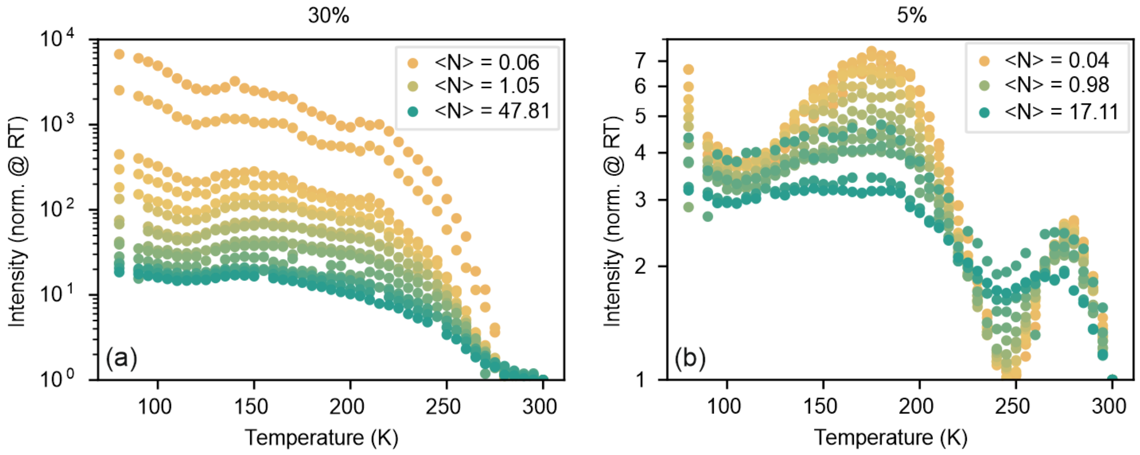

Figure S3.3 PLQY values for different fluences normalized at room temperature. Relative PLQY values for different fluences for (a) $C_{prec} = 30\%$ and (b) $C_{prec} = 5\%$ normalized at the RT value.

S3.4 Simulation of Poisson distributed excitation scenarios

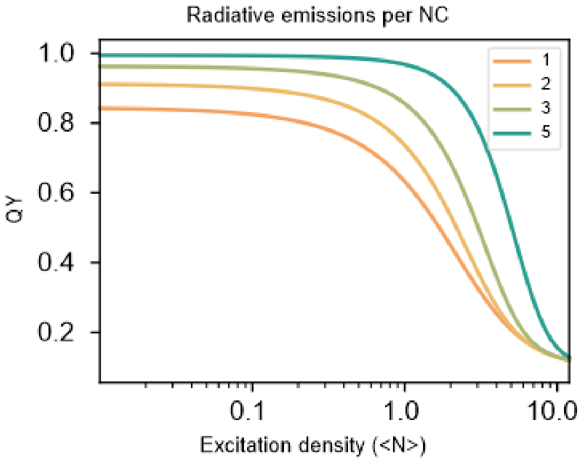

Figure S3.4 Simulated PLQY for Poisson distributed excitation densities. PLQY curves for different threshold levels of radiative recombinations per QD per excitation pulse ranging from 1 to 5 radiative emission per QD.

The probability of an individual QD to absorb an incoming photon can be described with a Poisson distribution. [7] The fluence onset where the PLQY starts to decay depends on the number of permitted radiative emission processes $Recomb._{rad}$ per excitation pulse in each QD. If the number of absorped photons is higher than $Recomb._{rad}$, the PLQY starts to decrease as simulated in **Fig S3.4**.

S4 Size distribution and charge carrier funneling effects

S4.1 Spectral analysis at different temperatures and fluences

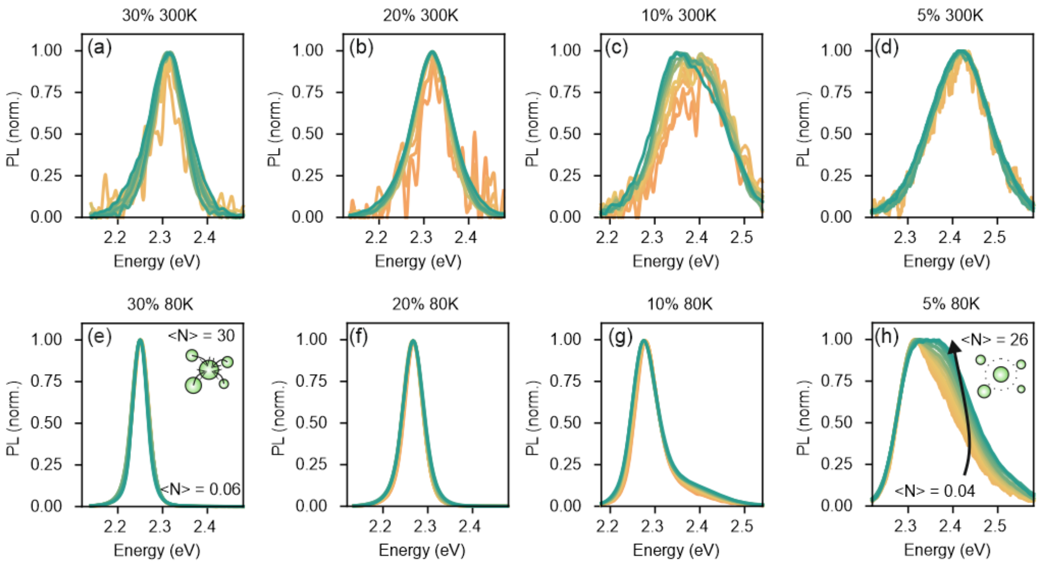

Figure S4.1 Emission spectra of samples with different C_{prec}. (a)-(d) RT normalized emission spectra. A gradual increase in peak width can be observed from 30% to 5 %. (e)-(h) Normalized emission spectra at low temperature. All graphs show excitation densities ranging from 0.04 (orange) to 30 <N> (green). A strong increase in FWHM can be observed in the 5% case. Insets show the expected charge funneling scenario in each case.

The emission bandwidth of interconnected ($C_{prec} = 30\%$) is narrower than the the isolated ($C_{prec} = 5\%$) case. This observation becomes more evident when considering emission spectra at 80K, where thermal broadening is reduced. In the interconnected case, the narrow spectral width of the PL can be rationalized considering a charge funneling process, where emission occurs from the energetically lowest site of the system. In contrast, in the isolated case, the emission occurs also from higher energetic states, as a funneling to the lowest state is prohibited. In addition, at 80K a fluence dependence of the emission spectrum becomes evident in the $C_{prec} = 5\%$ sample. This is interpreted as a higher absorption probability of larger QDs with lower emission energy, as their spatial absorption cross section is larger than the spatial absorption cross section of smaller ones. This higher absorption probability leads to an enhanced emission weight of larger QDs at low fluences. As fluence increases, the radiative emission of large QDs saturates with excess charge carriers recombining non-radiatively, whereas smaller ones continue to receive a larger load of excitations that are emitted radiatively. This mechanism further evidences the isolated character of the $C_{prec} = 5\%$ sample.

S4.2 Energy resolved state filling dynamics evidenced by TAS

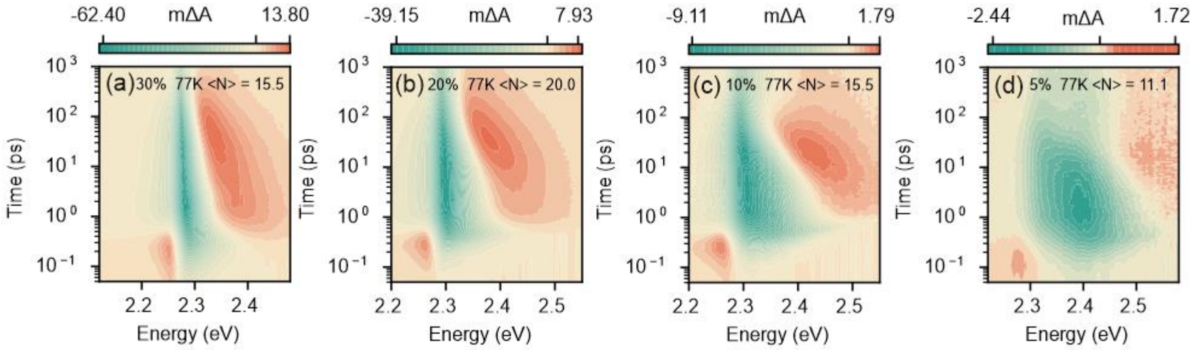

Figure S4.2 Transient absorption colormaps for different samples with different C_{prec}. False color maps of the change in absorption (ΔA) at excitation intensities well above <N>=1 at 77K.

References:

[1] Brus, L. Electronic Wave Functions in Semiconductor Clusters: Experiment and Theory. *J. Phys. Chem. A* **1986**, *90*, 2555– 2560

[2] Rubino, A.; Anaya, M.; Galisteo-López, J. F.; Rojas, T. C.; Calvo, M. E.; Míguez, H. Highly Efficient and Environmentally Stable Flexible Color Converters Based on Confined $CH_3NH_3PbBr_3$ Nanocrystals. *ACS Appl. Mater. Interfaces* **2018**, *10*, 38334– 38340

3] Perumal,A.; Shendre,S.; Li, M.; Tay,Y. K. E.; Sharma,V. K.;Chen,S.; Wei,Z.; Liu, Q.; Gao,Y.; Buenconsejo,P. J. S.; et al. High Brightness Formamidinium Lead Bromide Perovskite Nanocrystal Light Emitting Devices. *Sci. Rep.* **2016**, *6*, 1–10

[4] Shockley, W.; Read, W. T. Statistics of the Recombinations of Holes and Electrons. *Phys. Rev.* **1952**, *87*, 835–842.

[5] van Driel, A. F.; Nikolaev, I. S.; Vergeer, P.; Lodahl, P.; Vanmaekelbergh, D.; Vos, W. L. Statistical Analysis of Time-Resolved Emission from Ensembles of Semiconductor Quantum Dots: Interpretation of Exponential Decay Models. *Phys. Rev. B* **2007**, *75*, 035329

[6] Schueller, E. C.; Laurita, G.; Fabini, D. H.; Stoumpos, C. C.; Kanatzidis, M. G.; Seshadri, R. Crystal structure Evolution and Notable Thermal Expansion in Hybrid Perovskites Formamidinium Tin Iodide and Formamidinium Lead Bromide. *Inorganic Chemistry*, **2018**, *57*, 695-701.

7] Klimov, V.I. Optical Nonlinearities and Ultrafast Carrier Dynamics in Semiconductor Nanocrystals. *J. Phys. Chem. B* **2000**, *104*, 6112-6123.
